# Force matching: motor effects that are not reported by the actor

**DOI:** 10.1007/s00221-024-06829-4

**Published:** 2024-04-23

**Authors:** Michał Pawłowski, Joseph M. Ricotta, Sayan D. De, Mark L. Latash

**Affiliations:** 1 Department of Kinesiology, The Pennsylvania State University, University Park, PA 16802 USA; 2 Institute of Sport Science, Department of Human Motor Behavior, Academy of Physical Education in Katowice, 72A Mikołowska St, Katowice, 40-065 Poland

**Keywords:** Force drift, Force perception, Referent coordinate, Coactivation, Bilateral effects

## Abstract

We explored unintentional drifts of finger forces during force production and matching task. Based on earlier studies, we predicted that force matching with the other hand would reduce or stop the force drift in instructed fingers while uninstructed (enslaved) fingers remain unaffected. Twelve young, healthy, right-handed participants performed two types of tasks with both hands (task hand and match hand). The task hand produced constant force at 20% of MVC level with the Index and Ring fingers pressing in parallel on strain gauge force sensors. The Middle finger force wasn’t instructed, and its enslaved force was recorded. Visual feedback on the total force by the instructed fingers was either present throughout the trial or only during the first 5 s (no-feedback condition). The other hand matched the perceived force level of the task hand starting at either 4, 8, or 15 s from the trial initiation. No feedback was ever provided for the match hand force. After the visual feedback was removed, the task hand showed a consistent drift to lower magnitudes of total force. Contrary to our prediction, over all conditions, force matching caused a brief acceleration of force drift in the task hand, which then reached a plateau. There was no effect of matching on drifts in enslaved finger force. We interpret the force drifts within the theory of control with spatial referent coordinates as consequences of drifts in the command (referent coordinate) to the antagonist muscles. This command is not adequately incorporated into force perception.

## Introduction

Unintentional force changes have been reported in a variety of experiments (reviewed in Latash 2021a, b, 2023). In particular, imagine that a person is asked to press with fingers of a hand on stationary force sensors in isometric conditions and produce a constant force level within a comfortable range (e.g., 20–30% of maximal voluntary contraction level) with the help of visual feedback, and then the feedback is removed. Typically, force drifts toward lower magnitudes with the time constant of about 10 s are observed (Vaillancourt and Russell 2002; Ambike et al. 2015). The actor reports that the force was kept constant even when the drift reaches ≈ 30% of the initial force level after 10–15 s of performance without visual feedback. These large perceptual errors suggest that the natural sensory feedback is not sufficient to ensure adequate force perception (see also Cuadra et al. 2020) and requires help from other sources.

**Fig. 1:**
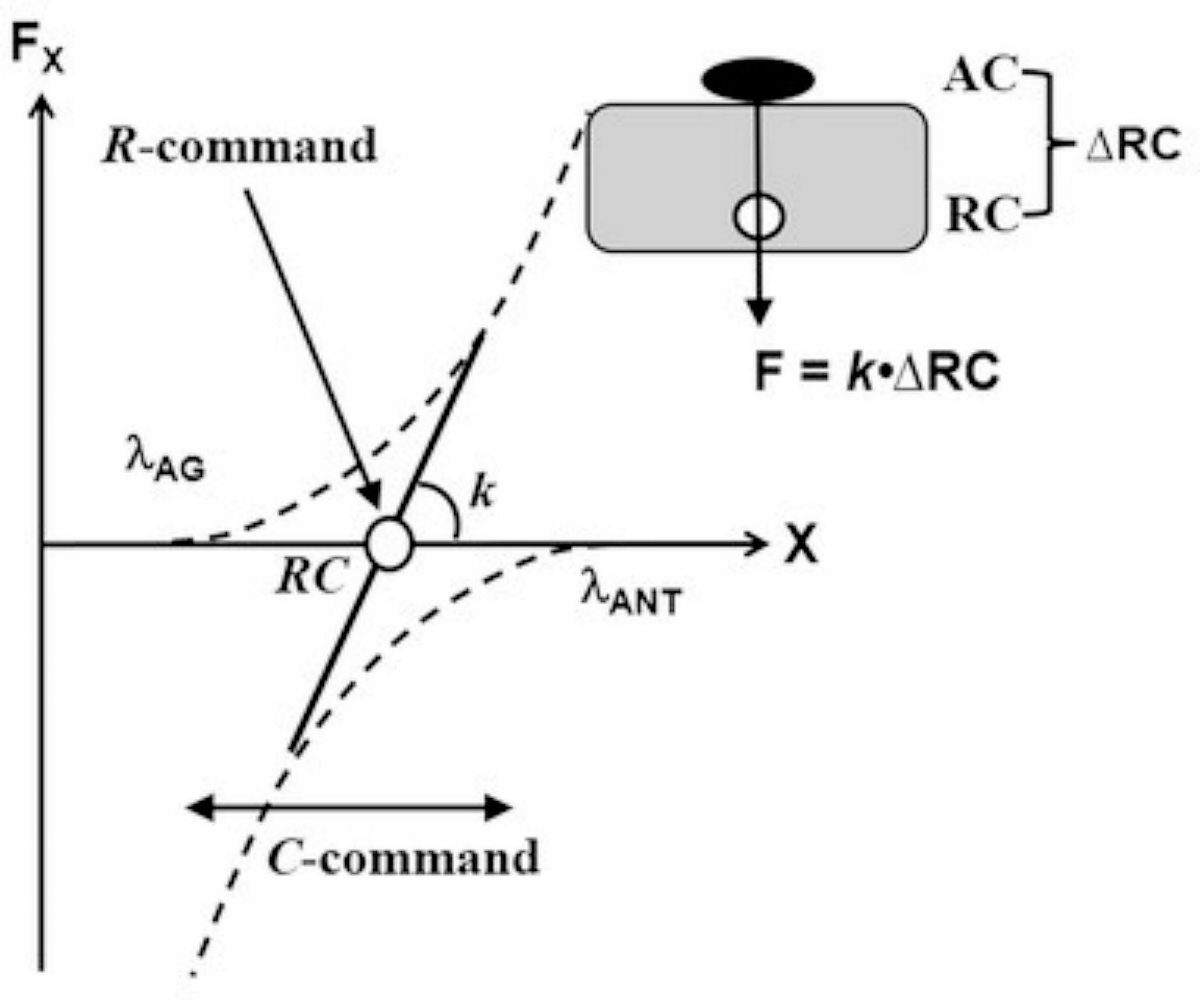
The control of an effector acting along a spatial coordinate *X* can be described with two force-coordinate characteristics (dashed lines) for the agonist and antagonist muscles generating positive and negative force (*F*_X_) values, respectively. The spatial location of the characteristics is defined by their spatial thresholds (λ_AG_ and λ_ANT_). Equivalently, the control can be described with two basic commands. The reciprocal command (R-command) defines the coordinate where F_X_ = 0, the referent coordinate (RC). The coactivation command (C-command) defines the spatial distance between λ_AG_ and λ_ANT_. Its changes are translated into changes in the slope (*k*) of the effector’s characteristic (the thick line). Force produced by an effector in isometric conditions is proportional to the difference (∆RC) between RC and actual coordinate (AC) and the apparent stiffness coefficient *k*

The observations of force drifts have been interpreted within the theory of movement control with spatial referent coordinates (RC) for the involved muscle groups (reviewed in Feldman 2015). Force production by an effector in isometric conditions is associated with setting commands to the agonist and antagonist muscle groups (λ_AG_ and λ_ANT_) that define their force-coordinate characteristics (dashed curves in Fig. 1). The overall effector force-coordinate characteristics is the algebraic sum of the muscle characteristics (thick line in Fig. 1). The control of the effector has been described with two basic commands. The reciprocal command (*R*-command) defines the coordinate where the resultant force of all muscles is zero (RC of the effector). The coactivation command (*C*-command) defines the spatial range between λ_AG_ and λ_ANT_; at the level of mechanics, the *C*-command defined the slope of the effector force-coordinate characteristic, i.e., its apparent stiffness (*k*, in Fig. 1). The force magnitude is defined by both the distance between RC and the actual effector coordinate (∆RC in Fig. 1) and *k*: F = *k*•∆RC. Unintentional force drifts have been interpreted as consequences of all natural systems to drift toward preferred states, typically states with minimal potential energy (Latash 2019, 2021, b). A number of earlier studies have suggested that unintentional drifts in force are associated primarily with drifts in the *C*-command, which is not incorporated adequately into force perception (Reschechtko and Latash 2017; Cuadra et al. 2021a, b).

Several studies of unintentional force changes used force matching by the contralateral hand to assess force perception (Abolins et al. 2020a; Cuadra et al. 2021a; Cuadra et al. 2021b). Under closer examination of Fig. 3 in Cuadra et al. (2021a), it becomes clear that the process of force matching by itself affected the ongoing force drift. The drift seems to stop at the time when the contralateral hand applies force. This puzzling phenomenon (not noticed by the authors) suggests that neural structures involved in force production by a hand can use the rather inaccurate force produced by the contralateral matching hand (see also Solnik et al. 2017) as a sensory cue replacing the missing visual information. In other words, the matching hand force could play a helpful role as a kinesthetic reference during matching task in the absence of the visual feedback. Note that the participants in the study by Cuadra et al. (2021a) did not report any force changes and were confident that they performed the original task and matched the actual force level accurately.

In general, bilateral interactions between homologous effectors are not unexpected given the well-known effects of bilateral inhibition and facilitation (Ohtsuki 1983; Vandervoort et al. 1984; Howard and Enoka 1991; Archontides and Dazey 1993; Oda and Moritani 1994; Ebben et al. 2009). Moreover, one of the earlier studies documented the so-called strong and weak interactions between the hands seen when a one-hand task turns into a two-hand task (Li et al. 2002). However, in all those studies, involving the contralateral effector led to deterioration in performance, while in the study of Cuadra et al. (2021a), the apparent stop of the unintentional force drift may be viewed as improvement in the task performance.

In the current study, we explored the effects of force matching on ongoing (unperceived) force drift. We used the standard paradigm whereby a participant produces a comfortable force level by pressing with a set of fingers with the help of visual feedback and then the feedback is removed. Our first question was: Can force matching by the contralateral effector stop the drift? Based on the cited figure from Cuadra et al. (2021a), we expected a positive answer (Prediction-1). In the cited study, matching was always done at the same time, 10 s after turning the feedback off, i.e., when the drift approached its maximal magnitude. We explored the effects of force matching happening at different times: Before turning the visual feedback off (i.e., before the drift started), early in the drift period, and late in the drift period. If force matching provides a sensory cue, we expected similar effects, i.e., force drift elimination independently of the matching time (Prediction-2).

We also explored whether effects of force matching could be seen in a finger that is not instructed to produce force. Note that when a subset of fingers produce force intentionally, other fingers of the hand produce force unintentionally (the phenomenon of enslaving, Zatsiorsky et al. 2000). In the absence of visual feedback, force by the enslaved fingers tends to drift toward higher magnitudes (Abolins et al. 2020; Hirose et al. 2020). These effects were interpreted as consequences of the spread of excitation over finger cortical representations. These phenomena are not of perceptual origin, and we expected no effects of matching on the force drift in the enslaved fingers (Prediction-3). Another exploration addressed the effects of hand dominance: The participants performed the task with either dominant hand or non-dominant hand and used the other hand for force matching. Effects of dominance could be expected based on earlier studies (larger drifts in the dominant hand, Park et al. 2012; De Freitas et al. 2019) in compliance with the dynamic dominance hypothesis (Sainburg 2005).

## Methods

### Participants

Twelve adults aged 23.75 ± 4.69 years, six females and six males, height: 1.73 ± 0.087 m; mass: 74.91 ± 8.86 kg (mean ± standard deviation) volunteered to take part in our study. All participants were right-handed (self-reported) in relation to daily activities such as writing and eating and free for any neurological and neuromuscular disorders that could affect the upper limbs. They had normal or corrected to normal vision. Participants gave written informed consent approved by the Office for Research Protections of the Pennsylvania State University and were naïve to the purpose of the study.

### Equipment

**Fig. 2:**
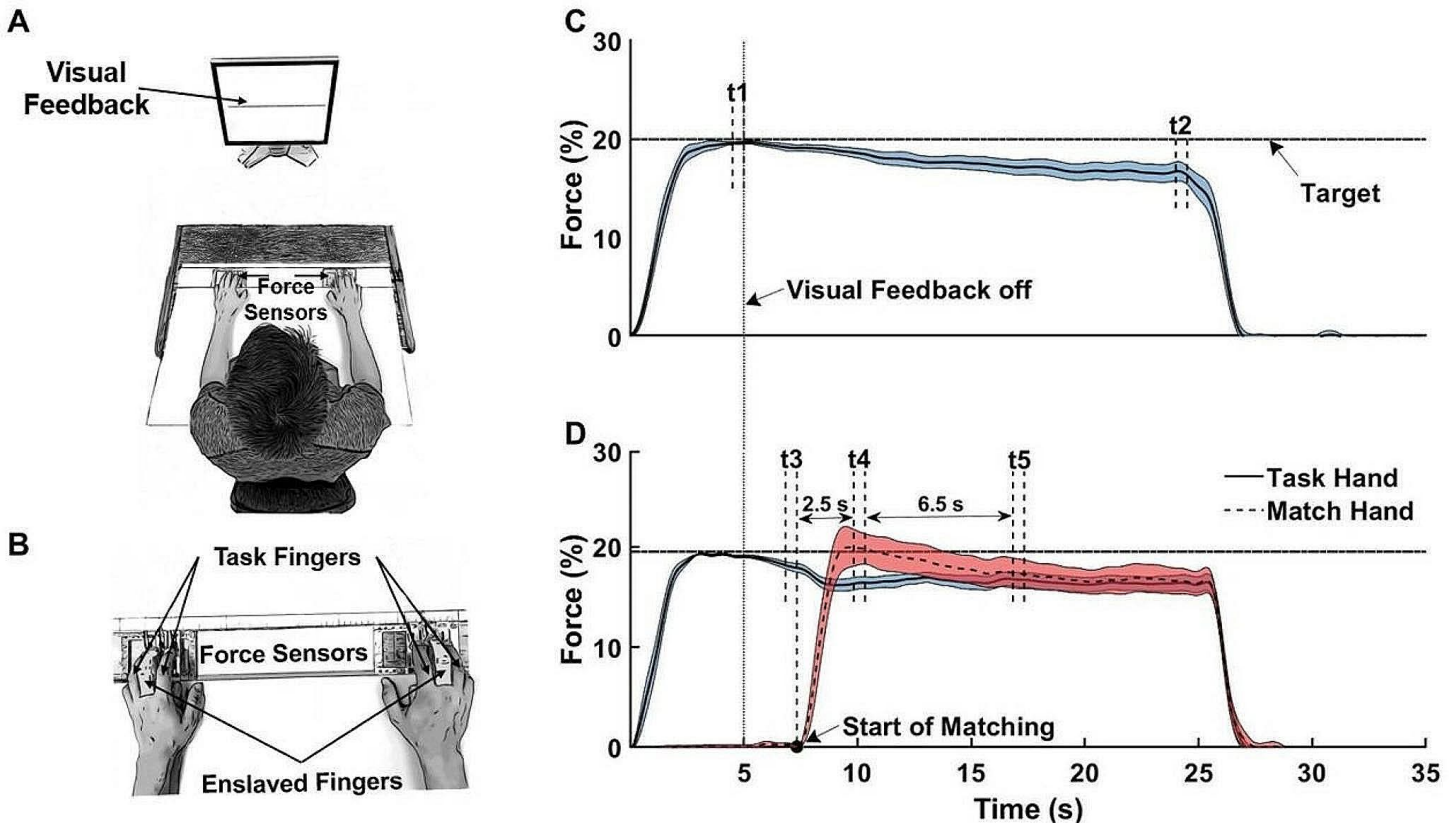
**A** A scheme of the experimental setup. **B** Position of the hands and fingers involved in the task. **C** The representative example of force production task without matching of the force time profile for the task hand: *t1* - time interval between 4.5 to 5 s. t2 - time interval between 24.5–25 s. **D** The representative example of matching task with the force time profiles for the task hand (solid line) and match hand (dashed line): *t3–0*.5 s before the beginning of matching; *t4* - time interval of 2.5 s after beginning of matching; *t5* - time interval of 9 s after matching

Six force/torque transducers (Nano-17 sensors, ATI Industrial Automation, Garner, NC, USA) were mounted in two rigid frames (140 × 90 mm each). Inside of each frame, there were three sensors used to measure the forces of the index (I), middle (M) and ring (R) fingers. The top of each sensor was covered with 320-grit sandpaper to prevent slipping. The sensors within each frame were spaced 3 cm in the mediolateral direction. In the anterior-posterior direction, position of the sensors was adjusted to match the individual finger anatomy and maintained throughout the testing session. The data acquisition and visual feedback provided to the participants were based on a customized LabView software. The signals from the force sensors were collected, amplified and sampled at 200 Hz with the two 16-bit cards (PCI-6225, National Instruments, Austin, TX). Feedback was presented via a 20” monitor placed 0.6 m in front of the participant at eye level. The experimental setup is depicted in panels A and B of Fig. 2.

### Procedures

The experiment involved one session in the laboratory and lasted between 1 and 1.5 h. Participants were asked to sit in a chair with the forearms laying comfortably on the table and the tips of the three fingers (I, M, and R) positioned on the sensors. The thumbs and pinkies rested naturally on the board. The hands rested on the dome-shaped wooden blocks, which helped to stabilize the position of the wrists and fingers. The protocol involved familiarization with the setup, maximal voluntary contraction (MVC) test, and the force production/matching main task. Across all tasks, participants were instructed to use only the I and R fingers, whereas the M fingers always remained on the sensors. The forces produced by all three fingers were recorded.

#### Maximal voluntary contraction (MVC) test

Participants were instructed to press on the sensors “as hard as you can” with the I and R fingers of each hand separately over the 3-s period followed by a 30-s rest interval. During this task, the visual feedback was provided as the sum of the forces of the two task fingers. The peak magnitude of the total force was used to normalize the main task.

#### Force production and matching task

In these trials, both hands could be involved, addressed further as the task hand and the match hand. The participants were instructed to press with the I and R fingers of the task hand and match the target shown on the screen as a red horizontal line. The target was set at 20% MVC force magnitude. Participants always got visual feedback as the sum of the I and R forces of the task hand (F_TASK_) during the first 5 s of each trial in each condition of the experiment. Then, the feedback on F_TASK_ stayed on the screen (feedback on) or disappeared (feedback off) and the participants were always instructed to keep pressing with the same magnitude of F_TASK_ for 20 s more, until the trial ended. The participants were instructed to match the current magnitude of F_TASK_ by pressing with the I and R fingers of the match hand (F_MATCH_) using both visual (text on the screen) and verbal (by the experimenter) prompts at different matching times (see later). No visual feedback was ever presented for the match hand force. Both hands continued to produce force until the end of the trial. There were 10-s rest periods after each trial.

The instruction to match F_TASK_ produced by the task hand was given 4, 8 and 15 s from the beginning of the trial. Note that visual feedback on the task hand F_TASK_ was available during matching at 4 s, and it was removed during matching at 8 s and 15 s. The 4-s matching time condition may be viewed as a control for possible effects of force matching on the course of force drifts in the absence of visual feedback when the subjects kept the task-hand force unchanged during the addition of the other (match) hand. There was also a control condition when no force matching was required. Different matching time conditions were used randomly. The dominant and non-dominant hands were used as the task hand in a block-randomized design across participants. Six trials were performed for each of the matching time conditions.

After the experimental session, the participants were asked whether they had followed the instructions successfully at all times. Note that the instruction included explicitly keeping the force by the task-hand constant throughout the trial and matching it accurately. All participants expressed confidence in having performed the task correctly, which was consistent with previous studies (Vaillancourt and Russell 2002; Ambike et al. 2016b; Parsa et al. 2016, 2017; Reschechtko and Latash 2017) where participants did not report the observed force drifts.

### Data analysis

The force signals were low-pass filtered at 10 Hz using a 4th -order zero-lag Butterworth filter. F_IR_ was computed as the sum of the vertical forces produced by the I and R fingers in each hand. Force changes during the no-feedback conditions were quantified using the following indices.

First, the total force drift magnitude in the task hand (∆F_TASK_) was calculated as:

1 $$\varDelta {F}_{TASK}=\frac{{F}_{TASK}\left({t}_{2}\right)-{F}_{TASK}\left({t}_{1}\right)}{{F}_{TASK}\left({t}_{1}\right)}$$

where F_TASK_(t_2_) and F_TASK_(t_1_) are the magnitudes of F_TASK_ averaged over 500-ms time windows before removing visual feedback, t_1_: {4.5; 5} s, and at the end of the trial, t_2_: {24.5; 25} s, respectively (see Fig. 2C). Positive values of ∆F_TASK_ corresponded to increases in force, whereas negative values corresponded to decreases in force over time. Here and later, the 500-ms time windows were selected based on pilot and previous studies (e.g., Reschechtko and Latash 2017; Abolins and Latash 2022) as a pragmatic solution to avoid both amplifying data variability (for shorter time windows) and effectively averaging data over intervals of force drift (for longer time windows). This time window was used for all the main dependent variables.

Second, the magnitude of transient changes of F_IR_ in the task hand (∆F_TR_) immediately after the start of the matching episode was computed as:

2 $$\varDelta {F}_{TR}=\frac{{F}_{TASK}\left({t}_{4}\right)-TASK\left({t}_{3}\right)}{{F}_{TASK}\left({t}_{3}\right)}$$

where F_TASK_(t_4_) and F_TASK_(t_3_) are the magnitudes of F_TASK_ averaged over 500-ms time windows immediately prior to a matching episode (t_3_) and {2.5; 3} s after the matching started (t_4_), respectively (see Fig. 2D). The beginning of a matching episode was defined in relation to the matching time.

Third, the magnitude of force drift in the task hand was estimated over next 7 s of each matching episode (steady-state drift, ∆F_SS_), which was the longest time available over all the matching conditions:

3 $$\varDelta {F}_{SS}=\frac{{F}_{TASK}\left({t}_{5}\right)-{F}_{TASK}\left({t}_{4}\right)}{{F}_{TASK}\left({t}_{4}\right)}$$

where t_5_ is the magnitude of F_TASK_ averaged over the 500-ms time window starting 8.5 s after the beginning of the matching episode (Fig. 2D).

In addition, the magnitude of force drift (∆F_MATCH_) was computed in the match hand:

4 $$\varDelta {F}_{MATCH}=\frac{{F}_{MATCH}\left({t}_{2}\right)-{F}_{MATCH}\left({t}_{4}\right)}{{F}_{MATCH}\left({t}_{4}\right)}$$

where t_2_ is the magnitude of F_MATCH_ averaged over the 500-ms time window starting one second before the end of a trial in match hand (Fig. 2C and D). Furthermore, the maximal mismatch (∆F_PEAK_) between the forces produced by the match hand (F_MATCH_) and task hand (F_TASK_) was calculated early in the matching:

5 $$\varDelta {F}_{PEAK}={F}_{MATCH}\left({t}_{4}\right)-{F}_{TASK}\left({t}_{4}\right)$$

where t_4_ is the magnitude of F_IR_ over the 500-ms time window, started 2.5 s after beginning of matching (Fig. 2D). F_MATCH_ as well as F_TASK_ were normalized by the MVC test in the task hand since the task was always set in proportion to that value. Finally, ∆F_PEAK_ was expressed in percent. Positive values of ∆F_PEAK_ correspond to overshoots, whereas negative values reflected undershoots by the match hand.

The force magnitudes produced by the enslaved Middle fingers (F_M_) in both hands were analyzed. F_M_ in the task hand was normalized to the F_M_ average magnitude over the 500-ms time window before removing the visual feedback (t_1_) and fourth time window (t_4_) for the match hand (Fig. 2). The total force drift magnitude in the M finger (∆F_M_) in the task and match hand, the magnitude of transient changes of F_M_ in the task hand (∆F_TR_) and the magnitude of steady-state drift in F_M_ (∆F_SS_) were computed using the same computational steps as in Eqs. (3), (4), and (5). In addition, the index of enslaving (E) was calculated as:

6 $$E=\frac{{F}_{M}}{{F}_{TASK}}$$

and quantified over the same time intervals.

### Statistics

The values are reported in the text and figures as means ± standard errors unless mentioned otherwise. The normality assumption was checked with the Shapiro-Wilk test. Homogeneity of variance was checked with Levene’s test, and the sphericity assumption was checked with the Mauchly’s test. In case of sphericity violations, the Greenhouse-Geisser corrections for the degrees-of-freedom were used.

To test Prediction-1 in the Introduction, we tested the effects of force matching on the task hand F_IM_ drift magnitude (∆F_TASK_) with a 4 × 2 × 2 analysis of variance (ANOVA) with repeated measures with the factors *Matching Time* (four levels: Control, 4 s, 8 s and 15 s), *Feedback* (with and without) and *Hand* (dominant and non-dominant). To explore Prediction-2, we explored the immediate and steady-state effects of force matching on the task hand force, ∆F_TR_ and ∆F_SS_, with a 3 × 2 × 2 ANOVA with the factors *Matching Time* (three levels: 4 s, 8 s and 15 s), *Feedback* and *Hand*. Prediction-3 on drifts of the enslaved finger force (F_M_) was tested using similar ANOVAs on F_M_ and the enslaving index, *E*. Pairwise comparisons with Bonferroni corrections were used to explore significant effects. All statistical calculations were performed with Statistica 13 (Statsoft, USA) and Microsoft Excel 16 (Microsoft, USA). The level of significance was set at *p* < 0.05. All effect sizes are indicated as partial eta squared (η_p_^2^).

## Results

### Force drift in the task hand and effects of matching

**Fig. 3:**
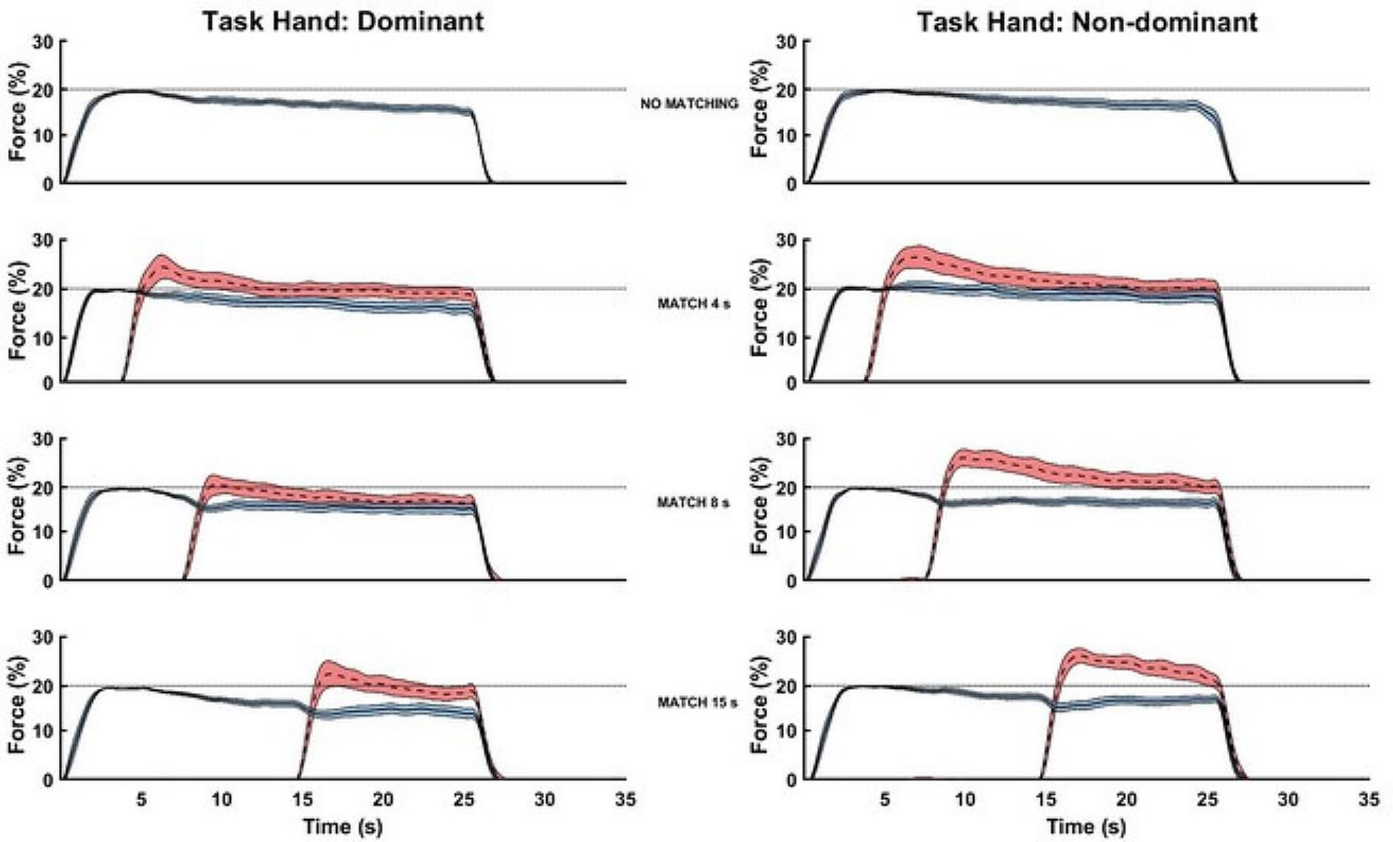
Time profiles of the forces produced by the instructed fingers (Index and Ring) together for all the experimental conditions. The horizontal dotted line represents the target force level. Mean time series across participants with standard error shades are shown. The task hand force is shown with the solid black lines (blue shadow), and the match hand force with the dashed lines (red shadow). Note the following consistent features: A transient overshoot and drift to lower values of the match hand force; a drift to lower values of the task hand force, which stops during the matching episode. The force axes are in percent of maximal voluntary contraction (MVC) force

Removing visual feedback of task hand force (F_TASK_) led to a consistent drift of F_TASK_ to lower force magnitudes. When the participants were asked to match the task hand force with the other hand, they showed a consistent transient overshoot followed by a drop in the match hand force closer to F_TASK_, but with a residual overshoot. This general pattern was consistent between trials using the dominant and non-dominant hands as the task hand. These patterns are illustrated as time series of F_TASK_ for each hand averaged across subjects with standard error shades in Fig. 3. Note that, during each matching episode, there is a visible trend for the F_TASK_ drift in the task hand to stop across all the matching time conditions. This trend is particularly pronounced during the matching later in the trial when visual feedback was turned-off, i.e., in the 8-s and 15-s matching conditions. The participants reported that they followed the instructions accurately, which included an explicit requirement to keep the force magnitude unchanged.

**Fig. 4:**
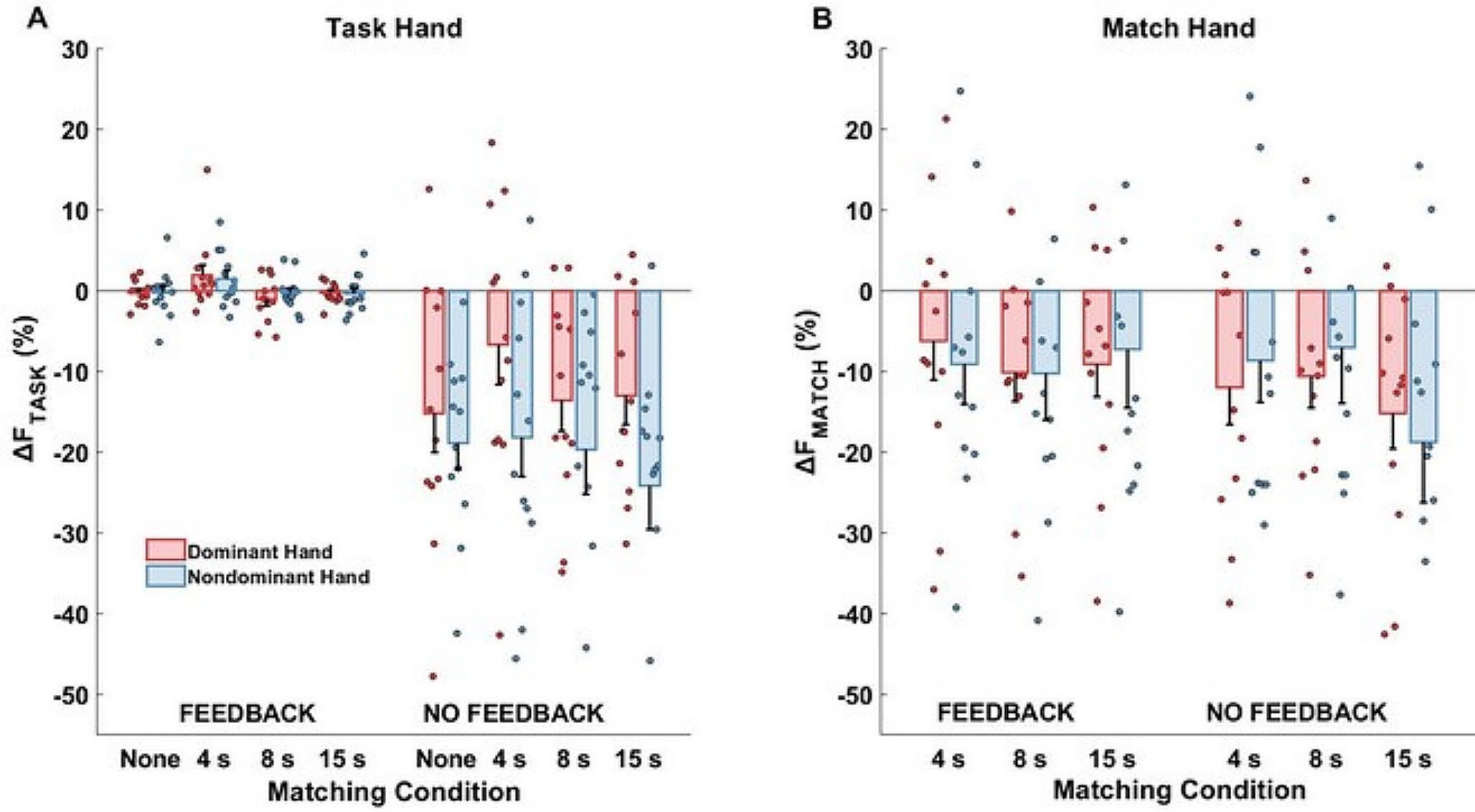
**A** The total force drift in the task hand. **B** The total force drift in the match hand. Means across participants are shown with standard error bars together with all the individual participant data points separately for the dominant (red symbols) and non-dominant (blue symbols) hands, for the four matching conditions. Note that visual feedback on the task hand force (“feedback”) eliminated the drift in that hand but had no effect on the match hand drift. The matching condition had no effect on the drift in either hand. The force axes are expressed in percent to the force magnitude within the t1 time interval, 4.5 s to 5 s, before removing the visual feedback

The total magnitude of F_TASK_ drift (∆F_TASK_) showed relatively minor changes across the matching conditions. This is illustrated in Fig. 4A, which shows the individual subject data as well as means with standard error bars. While the effect of *Matching- Time* was statistically significant (F_(3,33)_ = 2.967, *p* = 0.046, η_p_^2^ = 0.212), none of the pairwise contrasts reached the level of significance (*p* > 0.05). The average drift magnitude was similar between the dominant and non-dominant hands (*p* > 0.05). There was an expected effect of *Feedback* (F_(1,11)_ = 28.57, *p* < 0.001, η_p_^2^ = 0.722) with the drift across the no-feedback conditions being statistically significantly negative, while the drift in the feedback condition was, on average, close to zero.

**Fig. 5:**
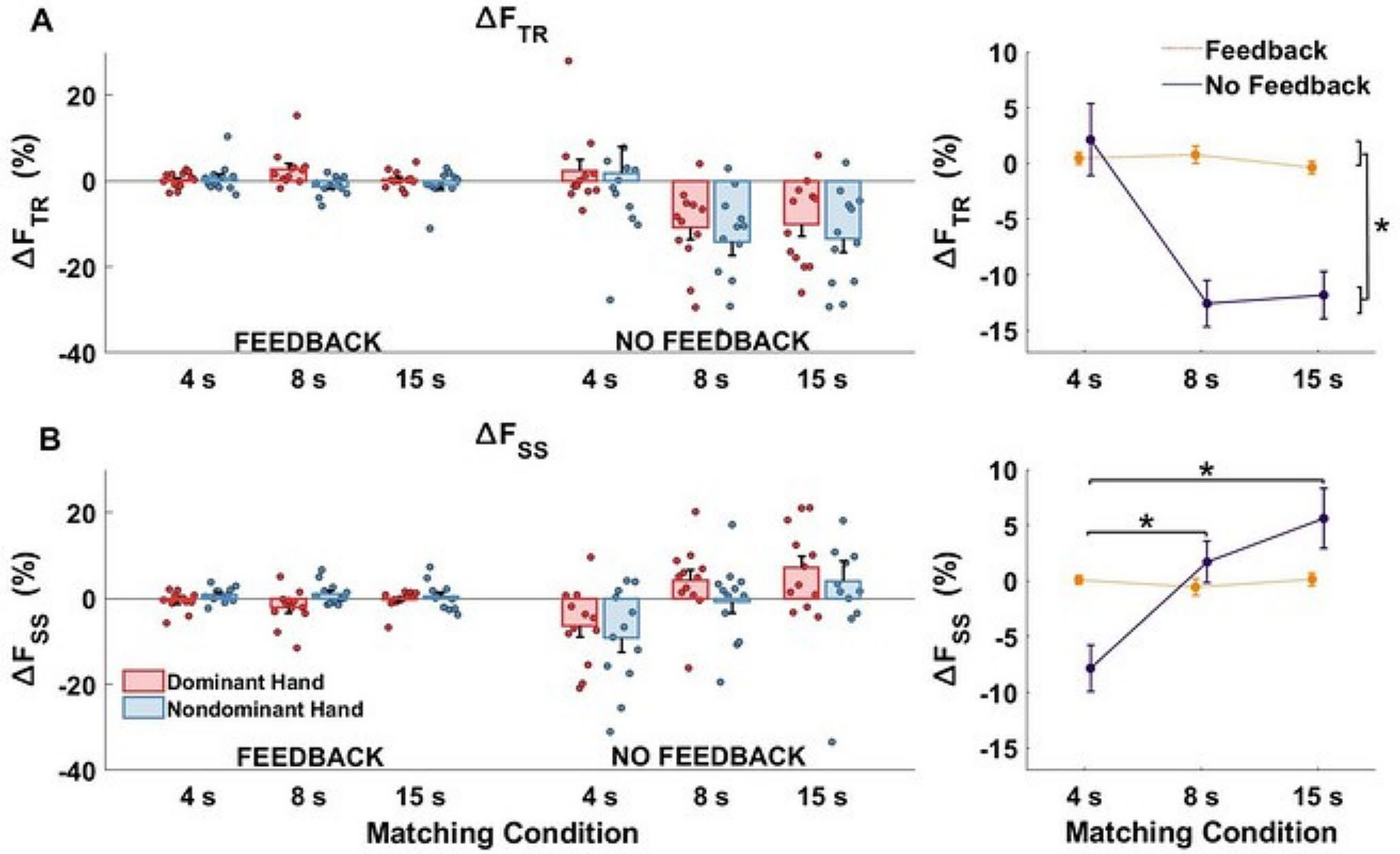
Force changes in the task hand across the feedback and matching conditions. **A** The transient drift force changes (∆F_TR_) across participants in task hand. **B** The steady-state drift force changes (∆F_SS_) across participants in task hand. The left panels show the means and standard error bars together with the individual participant data. The right panels illustrate the main results of the statistical analysis. Note the opposite trends of changes in ∆F_TR_ and ∆F_SS_ across the matching conditions while performing without visual feedback on the task hand force. The force axes are expressed in percent, explained the magnitude of drift change in relation to their initial magnitudes (t3 and t4 time intervals in transient and steady-state drift respectively)

A more detailed analysis revealed, however, that the similar magnitudes of ∆F_TASK_ across the conditions reflected two effects that nearly perfectly cancelled each other. These two effects were quantified with two indices reflecting transient acceleration of the drift in F_TASK_ early in the force matching episode (∆F_TR_) followed by F_TASK_ stabilization or even reversal during the new steady state (∆F_SS_) when both hands produced force. As a result, the sum (∆F_TR_ + ∆F_SS_) was close to the force drift magnitude in conditions without matching. The left panels in Fig. 5 illustrate these findings with both individual subject values and means with standard error bars. The three-way ANOVA on ∆F_TR_ showed statistically significant main effects of *Feedback* (F_(1,11)_ = 32.78, *p* < 0.001, η_p_^2^ = 0.746) and *Matching Time* (F_(1.34,14.74)_ = 9.301, *p* = 0.005, η_p_^2^ = 0.458). For both hands as the task hand, the magnitude of ∆F_TR_ was statistically significantly higher in the no-feedback condition (*p* < 0.001) and increased statistically significantly with an increase in the matching time. There were statistically significant differences between matching times of 8 s (*p* < 0.005) and 15 s (*p* < 0.005) as compared to the matching time of 4 s. There was also a statistically significant *Feedback* × *Matching time* interaction (F_(2,22)_ = 8.391, *p* = 0.002, η_p_^2^ = 0.433) reflecting the larger drop of F_TASK_ for matching at 8 and 15 s under the no-feedback condition only.

An opposite trend with changes in the matching time was observed for ∆F_SS_ (see the lower panels in Fig. 5). The three-way ANOVA on ∆F_SS_ confirmed a statistically significant effect of *Matching Time* (F_(2,22)_ = 8.157, *p* = 0.002, η_p_^2^ = 0.426). The drift towards lower force values for the 4-s condition disappeared for the 8-s condition (*p* < 0.05) and reversed its direction for the 15-s condition (*p* < 0.005). There was also a statistically significant *Feedback* × *Matching Time* interaction (F_(2,22)_ = 7.757, *p* = 0.002, η_p_^2^ = 0.428) reflecting the described pattern for the no-feedback condition but not for the feedback condition. No other effects were statistically significant.

### Force drifts in the match hand

**Fig. 6:**
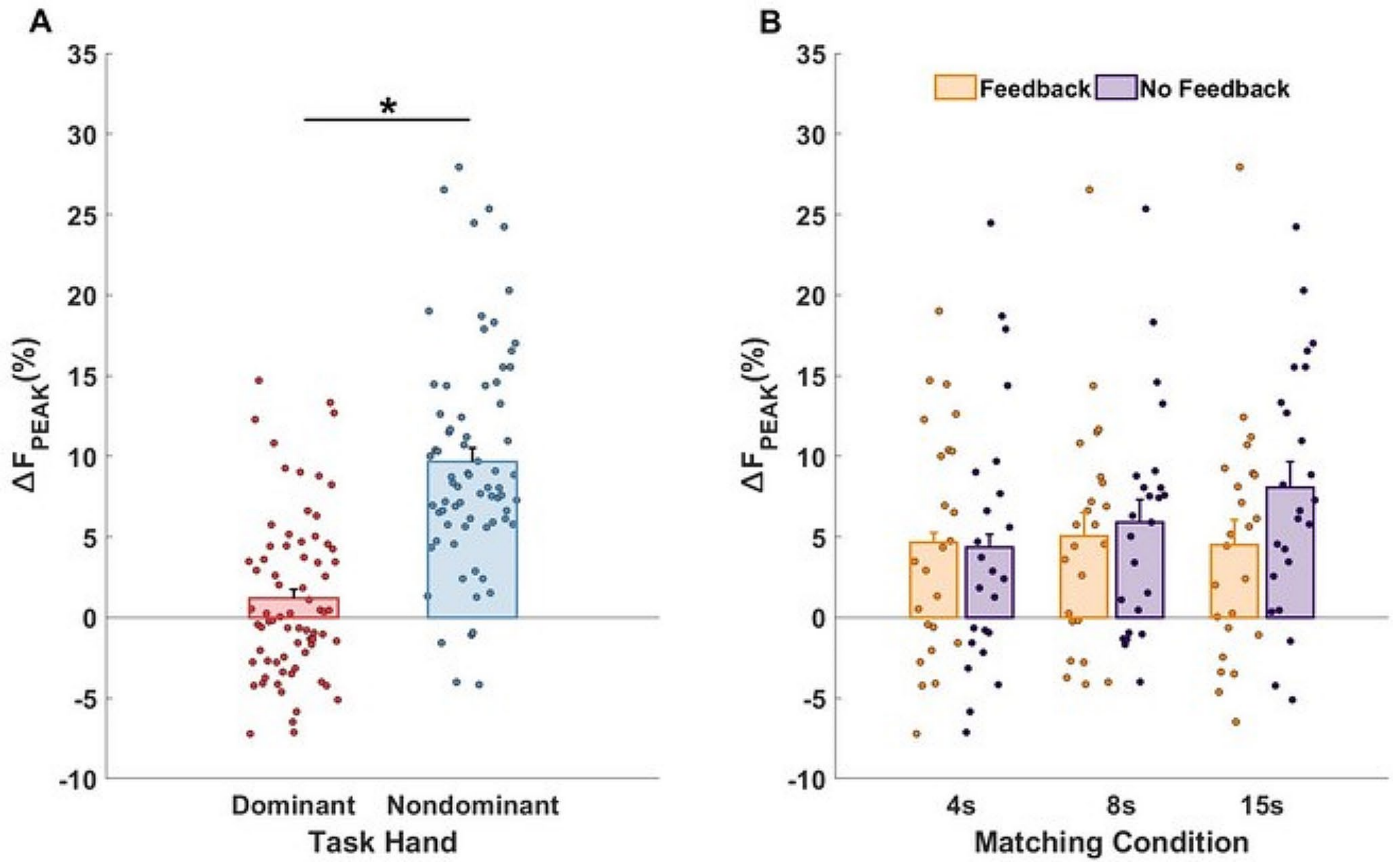
The force overshoot by the match hand across the participants and all experimental conditions. **A** The total overshoot by the match hand in relation to the hand dominance of the task hand. **B** The total overshoot in the match hand across the feedback and matching time conditions. Means across participants are shown with standard error bars together with all the individual participant data points. The data are expressed in percent of the MVC force magnitude by the task hand. Note the larger force overshoot when the matching was done by the dominant hand in the absence of effects of the feedback and matching conditions

As illustrated in Fig. 4B, there were consistent drifts in the force magnitude produced by the match hand toward lower values across all the matching conditions in both dominant and non-dominant hands. The magnitude of the drift (∆F_MATCH_) was, on average, − 11.14% ± 1.25 with respect to F_MATCH_ initial value in the t_4_ interval. ANOVA showed no statistically significant main effects and no interactions for the force drift magnitude in the match hand. Furthermore, all participants showed a consistent initial overshoot (∆F_PEAK_) of F_TASK_ by the match hand across the matching conditions. The three-way ANOVA showed a statistically significant effect of *Hand* (F_(1,11)_ = 17.41, *p* = 0.002, η_p_^2^ = 0.613). Performing the task with the non-dominant hand as the task hand caused larger overshoot by the dominant (match) hand (*p* = 0.002). This is illustrated in the left panel of Fig. 6 (see also Fig. 3). The right panel shows the overshoot values across the feedback and matching conditions.

### Effects of the enslaved finger force

The non-instructed Middle finger consistently produced non-zero pressing force (F_M_). The magnitude of this force showed consistent changes (∆F_M_) over the trial duration. In the task hand, ∆F_M_ showed a general trend to increase over the duration of the trial, on average by 29.07 ± 9.33% with respect to its initial magnitude quantified within the t1-time interval (see Fig. 2 in Methods). This increase was seen in both feedback and no-feedback conditions. There were no visible changes in ∆F_M_ that would be similar to ∆F_TR_ and ∆F_SS_ during the matching episode. Matching time had no statistically significant effect on ∆F_M_ (F_(3,18)_ = 2.015, *p* = 0.148).

In the match hand, ∆F_M_ was mostly negative with respect to its initial magnitude. On average, ∆F_M_ = − 22.09 ± 35.98%. Hand dominance (F_(1,5)_ = 0.413, *p* = 0.549) and matching time (F_(2,10)_ = 0.329, *p* = 0.727) had no statistically significant effects on ∆F_M_.

Analysis of the index of enslaving (*E*, see Methods) showed a general increase over the trial duration in both the task hand (by 52.72% ± 13.23) (F_(3,18)_ = 2.165, *p* = 0.127) and match hand (by 19.81% ± 16.34) (F_(2,12)_ = 1.825, *p* = 0.203) with respect to their initial magnitude (quantified in the time intervals t1 and t4, respectively). There were no effects of hand dominance and no effects of feedback.

## Discussion

We confirmed a number of earlier observations and addressed the specific predictions formulated in the Introduction with novel findings. In particular, in line with earlier studies, turning the visual feedback off the task finger force led consistently to slow force drifts to lower magnitudes (cf. Vaillancourt and Russell 2002; Ambike et al. 2015). At the same time, the non-instructed (enslaved) finger showed a consistent force drift in the opposite direction, toward higher magnitudes (cf. Hirose et al. 2020; Abolins et al. 2020). Force matching prior to removing visual feedback effectively turned the task into a two-hand task and led to similar force drift effects in the two hands after the feedback was removed (cf. Abolins et al. 2023). Overall, there was a tendency to overshoot the actual force with the matching hand with a transient and steady component (cf. Shergill et al. 2003; Voss et al. 2007; Walsh et al. 2011; Savage et al. 2015).

The results falsified the first of the specific predictions and supported the other two predictions. Indeed, force matching by the other hand did not stop the process of force drift, as introduced by prediction − 1, but accelerated it, albeit for a relatively brief time. These transient effects were followed by no additional force drift creating an impression that the drift had stopped (see Fig. 3). This impression, however, concealed the fact that the total amount of force drift was independent of the matching condition. In support of prediction − 2, the time of force matching had no significant effect on characteristics of total force drift. No significant effects of matching were observed in the enslaved finger force drifts as mentioned by prediction − 3.

Taken together, the results suggest that the force drifts reflect unintentional changes in one of the two basic neural commands, namely the coactivation command (the *C*-command, Feldman 1966, 1986, 2015). They also suggest that turning a one-handed task into a bimanual task is associated with transient loss of force stability, likely reflecting the addition of another level into the hierarchical scheme of control (Gorniak et al. 2007). Arguably, the most striking observation is that participants reported following the instructions (in particular, to keep the force magnitude constant) at all times in spite of the force drifts and matching effects. This observation has implications for mechanisms of force perception and points at limitations in incorporating changes in the *C*-command into kinesthetic perception (reviewed in Latash 2021a, b, 2023).

### The control of force production

Within the theory of control with spatial referent coordinates (RC) for the effector (reviewed in Latash 2010; Feldman 2015), force production tasks in isometric conditions are associated with specifying RC for the effector below the surface of contact using the reciprocal command (*R*-command) (Pilon et al. 2007). The difference between the actual effector coordinate and RC in spatial units is transformed into force units with the help of a stiffness-like coefficient (apparent stiffness, *k*, Latash and Zatsiorsky 1993) reflecting the coactivation command (*C*-command). This makes the control of the task abundant at the level of commands reflected in covarying changes in the *R*- and *C*-commands compatible with the desired force magnitude across repetitive trials (Ambike et al. 2016a; Reschechtko and Latash 2017).

Within this scheme, to produce a change in the force magnitude, the actor can use a change in the *R*-command and/or in the *C*-command. A hierarchical relation between the two basic commands was suggested (Levin and Dimov 1997; reviewed in Feldman 2015) with the *R*-command being hierarchically higher. According to this hypothesis, when a person performs a movement, the *R*-command defines the final state, and the *C*-command is transferred to the new spatial location. Recent studies of motor unit firing patterns within agonist-antagonist muscle pairs during force production tasks questioned the generality of this hypothesis (Madarshahian and Latash 2022). These studies documented grouping of motor units between the muscles into two major groups (MU-modes) with the composition reflecting directly the *R*- and *C*-commands. During cyclical force production, however, the MU-mode reflecting the *C*-command showed consistent modulation within the force cycle, whereas the MU-mode reflecting the *R*-command changed inconsistently across participants and, on average, showed no modulation with the force changes. These observations suggest that isometric force production tasks may be controlled primarily with changes in the *C*-command.

Within this theoretical framework, unintentional force drifts are caused by drifts in one of the two basic commands or in both commands. This interpretation of force drifts has been supported by measurements of the mechanical reflections of the *R*- and *C*-commands (i.e., RC and *k*, Ambike et al. 2016b; Reschechtko and Latash 2017, 2018) and by a study of force drifts against spring loads, which demonstrated smaller force drifts for more compliant loads (Abolins and Latash 2022).

This framework can also be used to address force matching. The abundance of solutions allows the matching hand to use different combinations of the *R*- and *C*-commands to reach about the same force level as the one by the task hand. Indeed, on average, the matching hand uses smaller magnitudes of the *C*-command and larger absolute magnitudes of the *R*-command (Abolins et al. 2020a) to generate the typical behavior with a relatively small overshoot of the force magnitude produced by the task hand (cf. Shergill et al. 2003; Voss et al. 2007; Savage et al. 2015; see Fig, 6). We will return to this scheme later in the Discussion to address implications of the findings for the processes of force matching and perception.

An alternative interpretation of unintentional force drifts invoked the concept of working memory (the “memory hypothesis”, Slifkin et al. 2000; Vaillancourt and Russell 2002). This hypothesis received indirect support in both clinical and brain imaging studies (Vaillancourt et al. 2003; Poon et al. 2012). It is not easily compatible, however, with the aforementioned study, which demonstrated smaller force drifts for more compliant loads (Abolins and Latash 2022). That study questioned the “memory hypothesis”, since the drifts did not define veridical or partly forgotten force magnitude changes, which also depended on the external loading conditions. It is also not clear to us how the “memory hypothesis” can handle the accelerated drift during the involvement of the matching hand and the observed. “floor effect” on the drift magnitude.

### Force matching: turning a one-hand task into a two-hand task

Force matching by homonymous contralateral effectors has been used in many studies of force perception (van Doren 1995, 1998; reviewed in Proske and Allen 2019; Proske and Gandevia 2012). This method has obvious problems related, in particular, to asymmetry of the effectors caused by effects of dominance (cf. Sainburg 2005). Another problem, which has not drawn as much attention is the fact that force matching effectively turns a one-handed force production task into a two-handed task. This is not a trivial transition, which is associated with strong interactions between the hands. In particular, when one hand tries to maintain a constant force level without visual feedback, starting slow ramp force production by the other hand leads to a rapid drop in the first hand’s force accompanied by a proportional unintentional rapid force increase by the second hand (Li et al. 2002). We observed similar effects in our experiment when the initiation of the matching episode led to a relatively fast force drop in the task hand and a transient overshoot by the match hand force.

Another effect of adding the other hand to a force production task is the disappearance of force-stabilizing multi-finger synergies in the first hand (Gorniak et al. 2007a), which may be causally related to the aforementioned rapid force drop. These effects have been discussed as reflections of a trade-off between synergies at different levels within a control hierarchy (Gorniak et al. 2007b, 2009). Indeed, a signature of a force-stabilizing synergy is a relatively large component of the inter-trial variance in the finger force space that does not lead to force changes, i.e., variance within the uncontrolled manifold for total force (V_UCM_, cf. Scholz and Schöner 1999; Latash et al. 2001). This component is larger than the variance component (V_ORT_) orthogonal to the UCM, which leads to changes in total force. Note, however, that large V_UCM_ in a two-hand task translates into large variance in the force of each of the hands. This produces large V_ORT_ if one analyzes synergies in the finger force space stabilizing each hand’s force. This qualitative analysis suggests that adding the matching hand to an ongoing force production task is expected to lead to a drop in the force stability, which adds a transient component to the slow force drift in the task hand observed in our experiment (see Fig. 5).

**Fig. 7:**
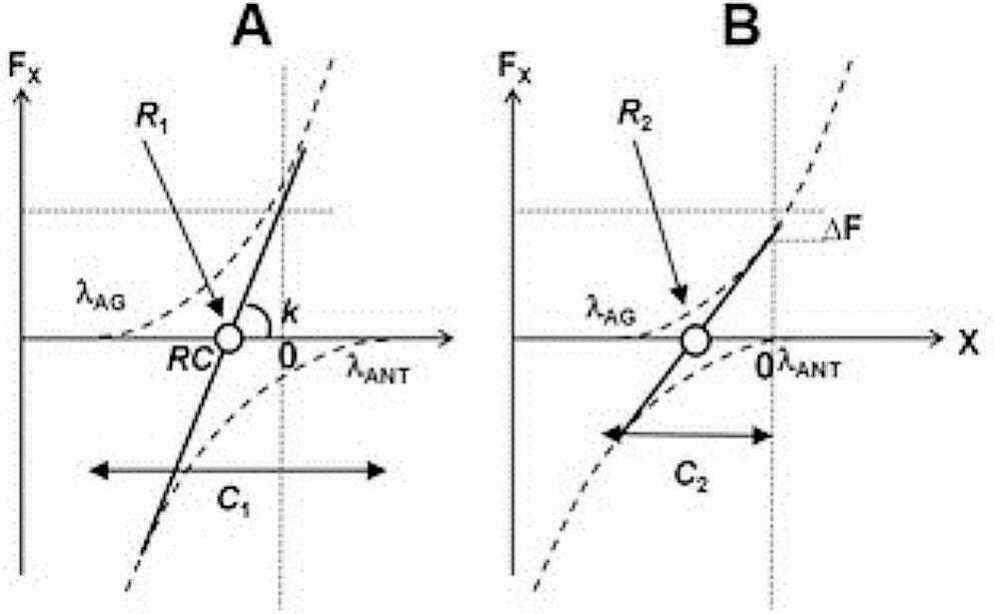
**A** In isometric conditions, the force magnitude is defined by the distance between the referent coordinate (RC, defined by the R-command) of the effector and its actual coordinate (shown by the vertical dotted line) and the apparent stiffness of the effector (*k*, defined by the C-command). See Fig. 1 for other explanations. **B** There are drifts of both λ_AG_ and λ_ANT_ toward the actual effector coordinate. The drift in λ_ANT_ is larger resulting in a drop in the magnitude of *k* and an increase in the absolute value of RC. When λ_ANT_ becomes equal to the actual coordinate, no mechanical changes or further drift can be seen

The processes contributing to the overall force change in the task hand are illustrated in Fig. 7. This figure shows the force-coordinate characteristics (the dashed curves) for the opposing muscles (agonist and antagonist) controlled by setting their thresholds, λ_AG_ and λ_ANT_, respectively (cf. Figure 1 in the Introduction). Equivalently, the control can be described with the *R*- and *C*-commands that define the overall effector force-coordinate characteristic (the thick line). Note that the main contributor to the force drift in the absence of matching is the slow drop in the *C*-command accompanied by a minor drift in the *R*-command, which partly compensates for the effect of the *C*-command drift on force magnitude (Reschechtko and Latash 2017). This process is naturally limited by the initial magnitude of λ_ANT_ and *C*-command. When λ_ANT_ reaches the actual effector coordinate (Fig. 7B), its further shift produces no effects on force, and net force is defined by λ_AG_ only. When the drift is accelerated by adding the matching hand, λ_ANT_ reaches the effector coordinate faster (during the transient phase) and further force drift becomes impossible. Of course, if the matching happens when the visual feedback on the task hand force is available, a quick correction based on the feedback eliminates the transient effects of matching, and the drift continues over the whole trial duration (see Fig. 3).

This interpretation of the origin of unintentional force drifts explains why, despite the apparent stop of the drift during the steady phase of force matching, its total magnitude is independent of the matching condition: The total drift magnitude is limited by the available range for the λ_ANT_ drift, i.e. by the initial level of coactivation of the antagonist muscle group. Matching can only accelerate reaching the limit of this range. The counter-directional effects of the force changes during the transient and steady-state phase of the matching episode (see Fig. 5 in Results) directly support this interpretation.

We observed only one significant difference related to hand dominance. The magnitude of the transient overshoot during the force matching was significantly larger when the task was performed by the non-dominant hand and matching – by the dominant hand. We see these observations as natural consequences of the dynamic dominance hypothesis of handedness (Sainburg 2005). According to this hypothesis, the non-dominant hand has an advantage in tasks that require steady-state performance in contrast to the dominant hand, which is better at performing tasks with quick changes in salient mechanical variables (supported by Zhang et al. 2006; De Freitas et al. 2019). Our matching task required steady force production by the match hand and, indeed, it was performed with smaller initial errors by the non-dominant hand when it served as the match hand.

### Lessons for force perception: perception-to-report and perception-to-act

Two roles of visual information have been established involving different brain pathways addressed as the ventral stream and dorsal stream (reviewed in Goodale and Milner 1992; Kravitz et al. 2011). They are related to creating a representation of the world and the actor in it and to guiding actions in that world. Under certain brain injuries, patients can show deficits in one of the streams only. For example, a patient can demonstrate severe neglect (reporting no visual experiences in one of the visual hemifields) and be able to act in that field, e.g., catch a ball thrown into that hemifield (Goodale et al. 1991).

Those observations can be summarized as differences between “perception-to-report” (an ability to describe perceptual changes to another person or to oneself, in real time or retrospectively) and “perception-to-act” (an ability to use sensory information to guide one’s actions). The latter concept is related to the concepts of direct perception and action-perception coupling within the field of ecological psychology (cf. Gibson 1979; Turvey 2007). For example, when we see an object, we can describe its properties (e.g., size, color, location, direction of motion, etc.), which is an example of “perception-to-report”. When a person stands and looks at a screen with a random dot pattern, motion of the points on the screen can induce postural deviations even if the person cannot report what exactly moved and how (e.g., Jeka et al. 1998).

A number of recent studies have suggested that somatosensory information can also play similar two distinct roles (cf. Dijkstra et al. 1994). In particular, active muscle coactivation has been shown to lead to an unintentional substantial increase in the pressing force (by about 50%), while the participants reported that the force had dropped somewhat (Cuadra et al. 2020). Along similar lines, a study of force perception by a finger involved in a multi-finger force production task led to different patterns when perception was estimated using force matching (action) by the other hand and when it was reported using a psychophysical scale (Cuadra et al. 2021b). In our study, similar to a number of earlier studies (Vaillancourt and Russell 2002; Ambike et al. 2016b; Parsa et al. 2016, 2017; Reschechtko and Latash 2017), the participants were confident that they followed the instruction to keep the force level by the instructed fingers unchanged. This happened in spite of both slow force drifts and faster, transient force changes during the matching episode.

Taken together, these observations suggest that kinesthetic perception has two components, perception-to-act and perception-to-report, likely involving different neurophysiological mechanisms. Perception-to-report clearly ignores the force changes induced by drifts in the neural commands, primarily in the *C*-command (see Fig. 7). Perception-to-act generates consistent errors in the contralateral hand overshooting the actual force magnitude (see also Shergill et al. 2003; Voss et al. 2007; Savage et al. 2015; Abolins et al. 2020a), and these errors are relatively consistent in conditions characterized by different magnitudes of the force drift (Cuadra et al. 2021a). In other words, errors in action lead to matching force magnitudes covarying with the actual force after the drift, i.e., force drift affects the matching force magnitude. This leads to a conclusion that perception-to-act mechanisms are sensitive to force drifts unlike perception-to-report ones.

A study of the two basic commands during force matching tasks in the absence of force drift (Abolins et al. 2020a) has shown that the matching hand consistently uses lower magnitudes of the *C*-command (and, correspondingly, larger absolute magnitudes of the R-command). Within our interpretation of the force drift phenomena, this allows expecting smaller drift magnitudes in the matching hand force since the drift magnitude is limited by the range afforded by the initial *C*-command level (see Fig. 7). This prediction has been supported in our study. Indeed, the task hand showed large force drift magnitudes without visual feedback, on average about 20% of the initial force level, while the average force drift in the match hand was, on average, only about 10%. Note that the typically larger initial force magnitudes produced by the matching hand (see Fig. 3) allowed expecting an opposite effect, i.e., larger force drifts in that hand (cf., Vaillancourt and Russell 2002; Ambike et al. 2015).

### Implications for mechanisms of finger enslaving

Finger interdependence, a well-known phenomenon (Schieber 1991; Li et al. 1998; Zatsiorsky et al. 2000), has been discussed as a reflection of both peripheral and central mechanisms (reviewed in Schieber and Santello 2004; Abolins and Latash 2021). The former includes the multi-digit extrinsic muscles and connective tissue links between fingers. The latter may get contributions from overlapping cortical representations of individual fingers and from diffuse reflex projections to alpha-motoneurons controlling the extrinsic muscles. A hypothesis on the brain origin of enslaving was suggested in the form of the “cortical piano” metaphor (Schieber 2001). According to this hypothesis, during everyday practice, humans develop coordinated inputs into the finger representations in the M1 cortex that produce proportional inputs to muscles and compartments serving individual fingers beneficial for the performance of functional tasks.

Patterns of finger enslaving have been viewed as robust and requiring extensive practice to be changed (e.g., Slobounov et al. 2002). However, a series of recent studies have provided evidence for relatively fast changes in enslaving during a single trial (Abolins et al. 2020a, b, 2023; Hirose et al. 2020). These studies have led to a formal representation of changes in force produced by enslaved fingers:

F_ENS_ = E_0_•η(t)•F_MAS_ (7).

where F_ENS_ and F_MAS_ stand for the enslaved and master finger force, respectively, E_0_ is the initial value of the enslaving coefficient, and η(t) is a monotonically increasing time function reflecting spread of excitation over the cortical finger representation. Our results confirm an increase in enslaving over the task duration in both hands as predicted by Eq. (7).

We failed, however, to see effects of adding the matching hand on the force of the Middle (enslaved) finger that would be analogous to the effects on the task finger force although such effects are expected from Eq. (7). This implies that the bilateral interactions, including the transient drop in the task finger force, reflect neural processes downstream of those reflected in Eq. (7) and affect RC commands to the task fingers but not to enslaved fingers. It is possible that the effects of matching on the force drift involve subcortical loops via the basal ganglia and cerebellum. This hypothesis is readily compatible with observations of larger force drift in patients with Parkison’s disease (Vaillancourt et al. 2001; Jo et al. 2016) and conclusions on the role of these circuits in ensuring stability of salient variables based on quantification of synergies across populations of neurological patients (reviewed in Latash and Huang 2015) and healthy persons at high risk for developing motor impairments induced, in particular, by occupational risk factors (Lewis et al. 2016).

One limitation of this study could be the chosen time resolution during the analysis, i.e. the 500-ms time window. This was a practical decision based on the cited earlier studies exploring slow force drifts. Force drifts do not exhibit perfectly smooth patterns and often show transients. As a result, in earlier studies, exponents were fitted to the averaged across trials force profiles (e.g., Ambike et al. 2015). The bilateral interactions associated with the addition of the contralateral hand are relatively brief (e.g., Li et al. 2002) and their analysis could benefit from better time resolution, smaller time windows. This may require a different experimental design to be a natural follow-up to the presented study.

To summarize, our results support two hypotheses in the field of force perception and production. First, they support the hypothesis on two distinct roles of kinesthetic information forming the bases for perception-to-act and perception-to-report. Second, they support the hypothesis that unintentional force drifts are consequences of drifts in the coactivation command naturally limited by its original magnitude. Taken together, these hypotheses form the core of the unified theory of movement production and perception based on changes in spatial referent coordinates for the involved effectors.

## Data Availability

The original data are available from the corresponding author at a reasonable request.
